# Does posttraumatic stress predict frequency of general practitioner visits in parents of terrorism survivors? A longitudinal study

**DOI:** 10.1080/20008198.2017.1389206

**Published:** 2017-11-20

**Authors:** Jon Magnus Haga, Lise Eilin Stene, Siri Thoresen, Tore Wentzel-Larsen, Grete Dyb

**Affiliations:** ^a^ Norwegian Centre of Violence and Traumatic Stress Studies (NKVTS), Oslo, Norway; ^b^ Institute of Clinical Medicine, Faculty of Medicine, University of Oslo, Oslo, Norway; ^c^ Department of Social Paediatrics, Division of Paediatric and Adolescent Medicine, Oslo University Hospital, Oslo, Norway; ^d^ Centre for Child and Adolescent Mental Health, Eastern and Southern Norway, Oslo, Norway

**Keywords:** Disaster, terrorism, parents, general practitioner, primary healthcare, posttraumatic stress disorder (PTSD), indirect exposure, child trauma, traumatization, unmet healthcare needs, Desastre, Terrorismo, Padres, Médico de cabecera, Atención sanitaria, Trastorno por estrés postraumático (TEPT), Exposición indirecta, Trauma infantil, Traumatización, Necesidades sanitarias no cubiertas, 灾难, 恐怖主义, 家长, 全科医生, 处理健康护理, 创伤后压力心理障碍, 间接暴露, 儿童创伤, 精神创伤, 未被满足的健康护理需求, • Posttraumatic stress reactions predicted increased frequency of early post-disaster GP visits in both mothers and fathers, suggesting that GPs play an important role in providing for post-disaster healthcare needs in parents and their families.• Distressed mothers of traumatized survivors may be at increased risk of being underserved in the delayed aftermath of a disaster, calling for attention from GPs.

## Abstract

**Background**: Life threat to children may induce severe posttraumatic stress reactions (PTSR) in parents. Troubled mothers and fathers may turn to their general practitioner (GP) for help.

**Objective**: This study investigated frequency of GP visits in mothers and fathers of adolescent and young adult terrorism survivors related to their own PTSR and PTSR in their surviving children.

**Method**: Self-reported early PTSR (4–5 months post-disaster) in 196 mothers, 113 fathers and 240 survivors of the 2011 Utøya terrorist attack were linked to parents’ three years pre- and post-disaster primary healthcare data from a national reimbursement claims database. Frequency of parents’ GP visits was regressed on parent and child PTSR, first separately, then in combination, and finally by including an interaction. Negative binominal regressions, adjusted for parents’ pre-disaster GP visits and socio-demography, were performed separately for mothers and fathers and for the early (<6 months) and delayed (6–36 months) aftermath of the terrorist attack.

**Results**: Parents’ early PTSR were significantly associated with higher early frequency of GP visits in mothers (rate ratio, RR = 1.31, 95%CI 1.09–1.56) and fathers (RR = 1.40, 95%CI 1.03–1.91). In the delayed aftermath, early PTSR were significantly associated with higher frequency of GP visits in mothers only (RR = 1.21, 95%CI 1.04–1.41). Early PTSR in children were not significantly associated with an overall increase in GP visits. On the contrary, in mothers, child PTSR predicted significant decrease in GP visits the delayed aftermath (RR = 0.83, 95%CI 0.71–0.97).

**Conclusions**: Our study suggests that GPs may play an important role in identifying and providing for parents’ post-disaster healthcare needs. GPs need to be aware that distressed individuals are likely to approach them following disasters and must prepare for both short- and long-term healthcare needs.

## Abstract (es)

**Planteamiento:** Las amenazas vitales a los niños puede inducir graves reacciones de estrés postraumático (PTSR, siglas en inglés de *posttraumatic stress reactions*) en los padres. Las madres y los padres preocupados pueden recurrir a su médico de cabecera (GP, siglas en inglés de *general practitioner*) en busca de ayuda.

**Objetivo:** Este estudio investigó la frecuencia de visitas al GP por parte de madres y padres de adolescentes y adultos jóvenes supervivientes del terrorismo relacionados con las reacciones de estrés postraumático propias y de sus hijos supervivientes.

**Métodos:** Los autoinformes de reacciones tempranas de estrés postraumático (4-5 meses después del desastre) en 196 madres, 113 padres y 240 supervivientes del ataque terrorista de Utøya de 2011 se enlazaron con los datos de atención primaria de salud de los padres (tres años antes y después del desastre) provenientes de una base de datos nacional de reclamaciones de reembolso. Se hizo una regresión de la frecuencia de las visitas al GP de los padres mediante regresiones binomiales negativas con las PTSR de los padres y el hijo, primero por separado, luego en combinación, y finalmente con la inclusión de una interacción. Los análisis se realizaron por separado para las madres y los padres con respecto a las consecuencias tempranas (<6 meses) y tardías (6-36 meses) después del ataque terrorista y se ajustaron para las visitas pre-desastre al GP de los padres y la sociodemografía.

**Resultados:** Las PTSR tempranas de los padres se asociaron significativamente con una mayor frecuencia temprana de visitas al GP en las madres (RR = 1,31, IC del 95%: 1,09-1,56) y padres (RR = 1,40, IC del 95%: 1,03-1,91). En las consecuencias posteriores, las PTSR tempranas se asociaron significativamente con una mayor frecuencia de visitas al GP en madres solamente (RR = 1,21; IC del 95%: 1,04-1,41). Las PTSR tempranas en niños no se asociaron significativamente con un aumento general de las visitas al GP. De hecho, en las madres, las PTSR del hijo predijeron una disminución significativa en las consecuencias tardías (RR = 0,83; IC del 95%: 0,71-0,97).

**Conclusiones:** Nuestro estudio sugiere que los médicos de cabecera pueden desempeñar un papel importante a la hora de identificar y la proporcionar las necesidades de atención médica de los padres después de un desastre. Los médicos de cabecera deben ser conscientes de que las personas angustiadas es probable que se acerquen a ellos después de los desastres y deben prepararse para las necesidades de salud a corto y largo plazo.

## Abstract (zh)

**背景：**孩子受到生命威胁后家长会产生严重的创伤后压力反应（PTSR）。困扰的家长可能会寻求全科医生(GP)的帮助。

**目标：**这个研究考察了处于青春期和成年早期的恐怖袭击幸存者的家长因为自身或者子女的PTSR而去探访全科医生的频率。

**方法：**2011年于特岛恐怖袭击中的196名母亲和113名父亲和240名幸存者的自我报告早期PTSR（灾难后4-6月），将这些自我报告与从国家索偿数据系统家长灾难3年之前和灾难之后的初级健康护理数据相联系。 先分别对家长探访全科医生的频率在家长与儿童的PTSR上进行负二项回归，再合并进行回归分析，最后检查了交互作用。恐怖袭击后的早期(<6个月)和后期（6-36月）的父亲母亲数据被分别进行检验分析，并控制了灾难前的家长探访GP次数和社会人口统计学变量。

**结果：**家长早期的PTSR显著地与母亲早期更高的GP探访率有关（rate ratio，RR=1.31，95%CI 1.09-1.56），也与父亲有关（RR=1.40，95%CI 1.03-1.91）。在灾难发生后期的比较中，早期PTSR只与母亲的高探访率有关（RR=1.21, 95%CI 1.04-1.41）。儿童的早期PTSR与GP探访率的整体增加无关。实际上，灾难发生后期儿童PTSR显著预测母亲探访率的下降（RR=0.83, 95%CI 0.71-0.97）。

**结论：**我们的研究表明全科医生在识别家长的创伤后健康关怀需求和提供帮助上有重要作用。全科医生需要知道在灾难后处于压力中的个体更可能探访，并且为应对短期和长期的健康关怀需要做好准备。

## 1. Background

Life threat to offspring may deeply affect their parents. Uncertainty of whether one’s child will live or die, the sense of powerlessness to protect a loved one and the fear of what comes next may leave harsh, lasting impressions in mothers and fathers of children of all ages, including parents of teens and young adults. The relief of reunion following a traumatic event may be accompanied by a second wave of emotional turmoil in parents: the shock of reconnecting with an injured, distressed and poorly-functioning child. Numerous challenges lie ahead for the traumatized family. Great responsibilities fall on the shoulders of parents. First, parents need to learn how to cope with their own stress reactions. Second, parents need to learn how to support their child under difficult circumstances. Third, practical challenges may arise as family members resume their daily lives, return to school, work and social arenas (Røkholt, Schultz, & Langballe, 2016). Adequate healthcare services may be critical in this demanding situation.

Parent traumatization through life threat to their child has mainly been studied in contexts of child’s chronic or acute illness (Cabizuca, Marques-Portella, Mendlowicz, Coutinho, & Figueira, 2009; Nelson & Gold, 2012) and sexual abuse (Dyb, Holen, Steinberg, Rodriguez, & Pynoos, 2003). High levels of parent posttraumatic stress reactions (PTSR) have consistently been reported. Few studies have addressed posttraumatic health in parents who learn that their offspring have been affected by disasters or terrorist attacks. One small study addressing 20 mothers of schoolchildren who survived the 2004 terrorist attack in Beslan reported high levels of PTSR, comparable to levels observed among the survivors themselves (Scrimin et al., 2006). A registry-based study of the aftermath of a pub fire in Holland in 2001 reported that mental and cardiovascular health problems, as recorded by the primary healthcare provider, were significantly more prevalent in parents of survivors with burns than in unaffected community controls (Dorn, Yzermans, Spreeuwenberg, & Van Der Zee, 2007). Thus, although limited, current evidence suggests that post-disaster ill-health in parents of disaster survivors may include both mental and somatic health problems that in turn may call for both mental and somatic post-disaster healthcare responses.

Our previous studies of the mothers and fathers of the 2011 Utøya terrorist attack survivors have demonstrated significantly elevated levels of early and lasting PTSR and depression/anxiety symptoms (Haga, Stene, Wentzel-Larsen, Thoresen, & Dyb, 2015; Thoresen, Jensen, Wentzel-Larsen, & Dyb, 2016). General practitioners (GPs) may play a key role in post-disaster management of parent mental and somatic healthcare needs, as a high number of individuals affected by a disaster may turn to their GP for help. Regardless of whether visits to the GP are related to the disaster or not, they allow GPs the opportunity to efficiently evaluate post-disaster healthcare needs.

Unmet healthcare needs are repeatedly reported following disasters (Brewin et al., 2010). A number of factors have been identified as barriers to post-disaster access to healthcare, including factors within the individuals in need (internal factors), as well as characteristics of the healthcare services or the community at large (external factors) (Kantor, Knefel, & Lueger-Schuster, 2016). Andersen’s behavioural model of healthcare service utilization (Andersen, 1995) has shaped much of current thinking on access to healthcare. The model divides predictors of healthcare consumption into three groups: (1) predisposing factors, including sociodemographic characteristics of the individual; (2) illness-related needs factors, including symptom severity and perceived needs; and (3) enabling factors, including availability of services, patient attitude towards health seeking and financial resources. The family context of post-trauma healthcare acquisition has previously solely been addressed in terms of assessing sociodemographic factors of family members (Elhai, North, & Frueh, 2005; Gavrilovic, Schützwohl, Fazel, & Priebe, 2005; Rodriguez & Kohn, 2008), largely with inconclusive results. Family context of illness-related needs factors and post-trauma healthcare acquisition have, to our knowledge, never been addressed. As PTSR of a parent and child may share a common aetiology, and thus frequently coexist and mutually influence or interact with one another, the family context of PTSR may prove an important factor in determining parents’ post-disaster GP-seeking behaviour.

In this study of the mothers and fathers of the 2011 Utøya terrorist attack survivors, we addressed frequency of parents’ post-disaster GP visits related to parents’ own PTSR and the PTSR experienced by their surviving adolescent or young adult child. Uniquely, the study combines registry-based longitudinal healthcare data before and after the attack, with self-reported distress in mothers, fathers and the survivors. Specifically, in regressions on parents’ post-disaster GP visits, adjusted for pre-disaster GP visits and a selection of hypothesized predisposing and enabling sociodemographic factors, we addressed the following research question: Did early parent or child PTSR predict frequency of post-disaster GP visits in mothers or fathers in the early (< 6 months) or delayed (6–30 months) aftermath of the Utøya terrorist attack?

## 2. Methods

### 2.1. Setting

The setting of this study is the 3-year post-trauma aftermath of the Utøya terrorist attack. In the summer of 2011, the youth summer camp on Utøya Island was attacked by an armed terrorist. For more than an hour, young campers were trapped with the perpetrator on the island, with no means of self-defence, limited access to shelter and scarce chances of escaping from the island. The shooting spree left 69 dead. Nearly 500 survived, of whom 35 sustained severe physical injuries (Bugge et al., 2015). Their families, all physically distant from Utøya, followed the development of the attack through live news coverage and intermittent telecommunication with their children on the island. In the aftermath of the attack, survivors of Utøya were reunited with their families across all regions of Norway.

Post-disaster healthcare for parents was provided by the regular healthcare system in Norway (Ringard, Sagan, Sperre Saunes, & Lindahl, 2013). Primary healthcare is the first-line provider of healthcare in Norway, making primary diagnoses, treating diseases, issuing sickness certificates, prescribing drugs and referring patients to specialist care as required. Primary healthcare, including emergency primary healthcare, is accessible throughout the country, available 24/7, publicly funded and is the mandatory entry-point for the publicly funded specialized healthcare services in Norway. Following the Utøya terrorist attack, a proactive outreach programme was established in affected municipalities, as a supplement to the regular services (Dyb, Jensen, Glad, Nygaard, & Thoresen, 2014; Haga et al., 2015). Crisis teams in all affected municipalities were to provide the initial psychosocial support. Dedicated contact persons were to monitor healthcare needs throughout the first-year post-disaster and to facilitate access to care as needed. A majority of the parents had a dedicated contact person in the municipality (69.1%, *n* = 206), of whom a majority was non-GP healthcare professionals (non-GP 89.1%, *n* = 180; GP 7.5%, *n* = 22; missing *n* = 4). A minority of the parents had a contact person who was also their regular GP.

### 2.2. Design

This study is a part of the Utøya study, a three-wave longitudinal observational study of the survivors and the parents in the 3-year aftermath of the Utøya terrorist attack. The current paper combines registry-based data on parental GP visits in the three years before and after the terrorist attack (22 July 2008 to 21 July 2014) with self-reported data by mothers, fathers and survivors at 4–5 months post-disaster.

### 2.3. Procedure

Recruitment to the study was based on police records of the survivors (*n* = 495). Survivors 13–33 years old (*n* = 482) were asked to name their caregivers. All caregivers nominated, henceforth referred to as parents irrespective of their legal, social or biological status, were invited to participate, along with their associated survivors. All invitations were distributed by mail and included information on the study and how to opt out. The survivors and the parents of the survivors born in 1992 or later were invited to participate in face-to-face interviews. Parents of older survivors (born in 1991 or earlier) and parents not available for interview participated through questionnaires. Interviews were conducted by trained healthcare professionals in the home of the informant or at an alternative place at the convenience of the informant.

The overall study comprised three waves of data collection. Waves 1 and 2 were open cohorts; invitations were extended to all survivors and their caregivers. Wave 3 was a closed cohort; only participants in at least one of the two previous waves were invited. This paper reports on parents that participated in both Waves 1 and 3, and on their respective child survivors. Data collection for Wave 1 commenced in early November 2011 and was largely completed (>95%) by mid-December 2011, five months after the attack. Wave 3 commenced in late March 2014, and was largely completed (>95%) by mid-July 2014, three years after the attacks. Registry-based healthcare data was collected from consenting participants following Wave 3.

All consents were provided in writing. The Regional Committees for Medical and Health Research Ethics in Norway approved the study.

### 2.4. Participation

Overall, 299 mothers and 233 fathers (*n* = 532 parents) participated in at least one of the three waves of the Utøya study, representing 68.7% (*n* = 331) of the survivors (13–33 years, *n* = 482). Two-thirds of the mother sample (*n* = 196, 65.6%) and half of the father sample (*n* = 113, 48.5%) met the inclusion criteria for this study (participated in Waves 1 and 3 and consented to sharing registry-based data), a majority of whom had participated through face-to-face interviews (mothers *n* = 152, 77.6%; fathers *n* = 88, 77.9%). Attrition in respect to the overall parent sample was significantly associated with male gender (OR 1.96, 95%CI 1.47–2.62, *p* < .001), but not with level of education, perceived financial status, country of origin, whether living alone/with a partner or PTSR, in adjusted regressions (Supplemental data Table 1a-b). All parents included in this study cared for one or more survivors of the terrorist attack. In a majority of the parent participants (mothers *n* = 190, 96.9%; fathers *n* = 108, 95.6%), one or more of their surviving children participated alongside. In the remaining 11 parents, the surviving children did not participate. The child survivor sample (*n* = 240) was gender balanced (female *n* = 118, 49.2%; male *n* = 122, 50.8%). No distinction was made between mothers and fathers parenting sons, daughters or both. Five parents additionally experienced the loss of a child to the terrorist attack (1.6%).

**Table 1.** Parent participants according to their own and their children’s posttraumatic stress.

	mothers	fathers
Variables	mean (*SD*)/*n* (%)	mean (*SD*)/*n* (%)
**Parental distress**		
(*n* = 196 mothers, *n* = 112 fathers^a^)		
PTSR score per item, mean (*SD*)	1.35 (0.76)	0.88 (0.63)
PTSD classification, *n* (%)		
– full (3 criteria satisfied)	18 (9.2)	3 (2.7)
– partial (2 criteria satisfied)	57 (29.1)	12 (10.7)
– no (1 or 0 criteria satisfied)	121 (61.7)	97 (86.6)
**Distress in the child^b^**		
(*n* = 190 mothers, *n* = 108 fathers^c^)		
Child PTSR score per item, mean (*SD*)	1.55 (0.69)	1.58 (0.67)
Child PTSD classification, *n* (%)		
– full (3 criteria satisfied)	16 (8.8)	12 (10.9)
– partial (2 criteria satisfied)	78 (41.5)	45 (40.9)
– no (1 or 0 criteria satisfied)	94 (50.0)	51 (48.2)

^a^Insufficient data on PTSR in *n* = 1 father.

^b^Mean PTSR score of the survivor sample (*n* = 219) was 1.54 (*SD* = 0.71), with full and partial PTSD classified in 21 (9.6%) and 85 (38.8%) survivors, respectively. The values are not directly comparable to the figures in Table 1, as not all survivors were represented by both a mother and a father. Furthermore, some of the survivors were siblings (parented by the same mother and father). In the case of siblings, the child with the higher PTSR score was included in the analyses.

^c^Missing data in *n* = 6 mothers and *n* = 4 fathers due to non-participation of their surviving child.

### 2.5. Measures

Primary healthcare consumption was assessed through the Health Economics Administration (HELFO) database of reimbursement claims to the national insurance scheme. The register contains all GP services provided in Norway as a part of the regular GP scheme. At the time of the Utøya attack, most people in Norway were part of the regular GP scheme (99.6%) (The Norwegian Directorate of Health, 2012). All GP contacts dating from 22 July 2008 to 21 July 2014 were excerpted, irrespective of whether services were provided at a GP clinic, through house calls, by regular GPs or locum GPs, within regular office hours or when on-call. Consultations with other professionals, including nurses, psychologists, therapists and counsellors, were not collected. A total of 13,419 GP contacts were identified, of which 42 (0.3%) were duplicates, i.e. referring to the same appointments (matching date, time, diagnosis, provider and mode of contact). Of the remaining 13,377 unique entries of primary healthcare services, 7212 (53.9%) were in-person consultations with a GP (GP visits). Non-in*-*person contacts, including telephone or mail correspondence between patient and GP, administrative renewal of sickness certificates, communication between the GP and other healthcare providers or the Norwegian Labor and Welfare Administration, were not included. Data on admittance and discharge of the participants from hospitals in Norway was collected from the Norwegian Patient Registry (NPR).

PTSR of the past month, recorded at Wave 1 in both parents and survivors, were evaluated by the 20-item University of California, Los Angeles Posttraumatic Stress Disorder Reaction Index (UCLA PTSD-RI) (Steinberg, Brymer, Decker, & Pynoos, 2004). The questions were formulated to explicitly relate to the terrorist attack. Responses were recorded on a five-point Likert-type scale, ranging from 0 (never) to 4 (most of the time). Three symptoms were presented to the participants in terms of two alternatively formulated questions. The question attracting the higher frequency score was used in further analyses, reducing the number of items to 17. First, continuous PTSR average scores of the 17 items were calculated (possible range 0–4). Second, individuals with a probable posttraumatic stress disorder (PTSD) were identified by using the DSM-IV diagnostic criteria (American Psychiatric Association, 1994). Criterion A (exposure) was satisfied in all participants of this study. Criteria B (intrusion), C (avoidance) and D (arousal) were derived by grouping items related to these categories. Scores of 3 (often) and 4 (most of the time) were taken to indicate clinical complaints. If all diagnostic criteria were satisfied (i.e. ≥1 item for criterion B, ≥3 items for criterion C and ≥2 items for criterion D), parents were classified with full PTSD. Falling short of a full diagnosis, parents who satisfied two criteria were classified with partial PTSD. Missing values ≤25% within each sub-score were resolved by calculating mean scores of the remaining items. For one individual who had >25% missing values, PTSR were not assessed. Cronbach’s α of the total scale was 0.89–0.93 for the three subsamples investigated (mothers, fathers, survivors).

Socio-demography was collected at Waves 1 and 3 and included gender, age (at the time of the attacks), country of birth, level of education (none, primary, secondary, vocational or higher education/university degree) and whether living alone or with a partner (at the time of Wave 1). Financial status was assessed on a five-point Likert-type scale. The parents rated financial status relative to the general population, ranging from 1 (‘much better’) to 5 (‘much worse’). The responses were dichotomized into ‘average or better’ and ‘below average’. Discrepancies between the waves were resolved by using the first value reported.

### 2.6. Statistics

In this paper, posttraumatic stress is reported both on a continuous scale (mean PTSR score, 0–4) and as a likely diagnosis (no, partial, full PTSD). Parents’ primary healthcare consumption is reported as observed frequency of GP visits and as estimated rate ratio (RR) of GP visits based on regressions described below. Missing values remained <5% throughout the paper and are noted.

*Regressions*. Frequency of parents’ post-disaster GP visits was evaluated though negative binomial regressions (NB) (Elhai, Calhoun, & Ford, 2008). NB is a generalized linear regression model, in which the dependent variable is counted within a given time period (e.g. number of GP consultations within a year). A key feature of NB is that it may accommodate overdispersion in count data, i.e. variance greater than mean value, as was observed in our dataset. Separate analyses were performed in mothers and fathers and for the early (<6 months) and delayed (6–36 months) aftermath of the Utøya terrorist attack. PTSR measured at 3–5 months post-disaster (Wave 1) was assumed to reflect early levels of distress and hence assumed to validly serve as proxy for ‘early PTSR’. In parents caring for more than one surviving child, the child with the more severe PTSR was included in the regressions. In all regressions, PTSR were included on a continuous scale (mean PTSR score).

**Figure 1.**
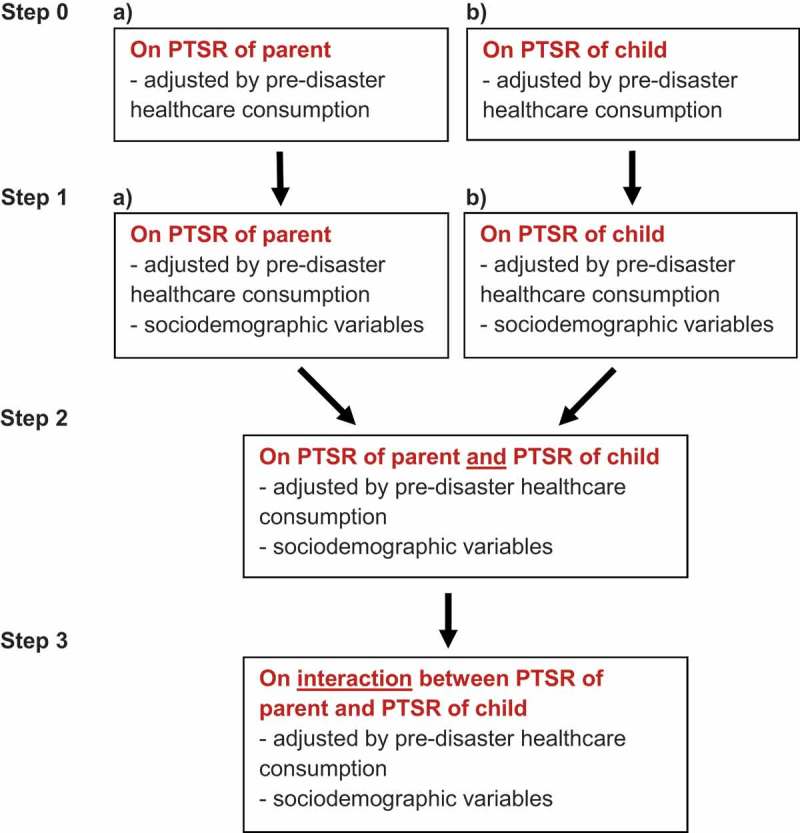
Overview of hierarchical regressions for parent primary healthcare consumption (frequency of GP visits).

Regressions were performed hierarchically (Figure 1). In all models, parents’ GP visits were the outcome measure and their 3-year pre-disaster GP visits were adjusted for. First, we included as predictors the parents’ early PTSR and, in a separate model, their child’s early PTSR. Second, parents’ sociodemographic status was included in the two models. Third, a model was assessed where both parent and child PTSR were included as well as the parents’ sociodemographic status. Finally, the interaction between parent and child PTSR was added.

Regressions on count data needs to be offset for the logarithm of the persontime at risk, often referred to as the observational period. In our model, hospitalization was considered to make an individual unavailable for care from a GP. Thus, observational period was defined as days of non-admission to a healthcare institution. Null hypotheses were rejected at *p* < .05.

Analyses were performed with R-version-3.1.2 (R Foundation for Statistical Computing), with the R packages MASS (7.3–45) for negative binomial regressions and boot (1.3–13) for bootstrap analyses.

## 3. Results

The mothers (*n* = 196) and fathers (*n* = 113) participating in this study parented one (*n* = 297, 96.1%), or more (n=12, 3.9%) survivors of the Utøya terrorist attack. Mean age (at time of attack) was 46.7 years (*SD* = 5.8) in mothers and 49.7 years (*SD* = 5.9) in fathers. Mean age (at time of attack) of their associated participating survivor (*n* = 240) was 18.5 years (*SD* = 2.7). A majority of the parent participants cohabited with a partner (*n* = 244, 79.7%, missing *n* = 3) and rated their financial status as average or above (*n* = 251, 81.2%). The parents were well educated (higher education, *n* = 187, 60.5%) and largely of Norwegian origin (*n* = 283, 91.6%).

PTSD classification of the parents and the PTSD classification of their surviving children are presented in Table 1. Notably, more than one in three mothers and one in eight fathers were classified with full or partial PTSD in the early post-disaster period. Furthermore, half of both mothers and fathers were found to care for a survivor classified as having either partial or full PTSD.

**Figure 2.**
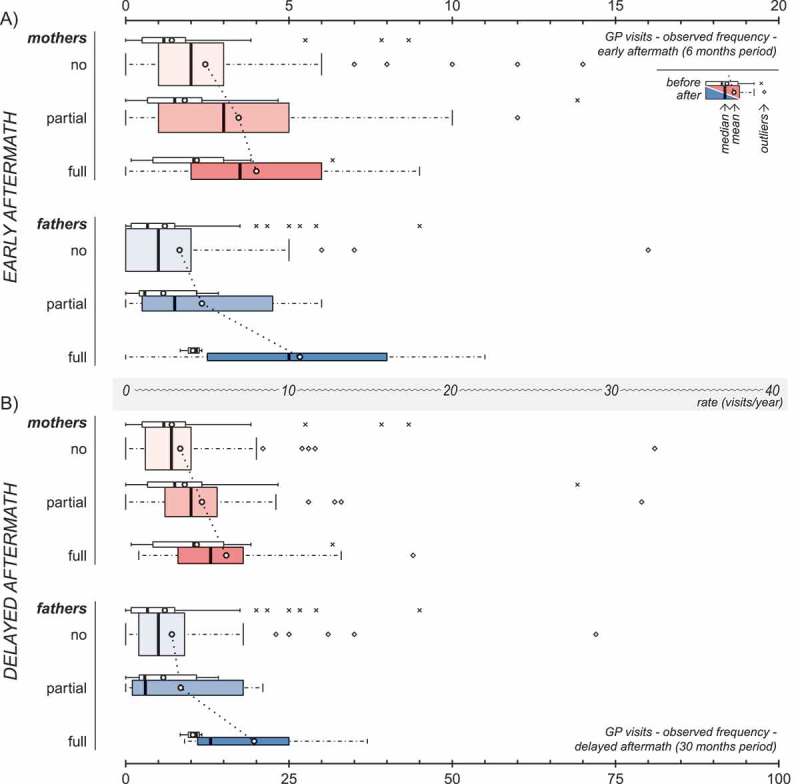
Observed post-disaster frequency of GP visits in mothers (red) and fathers (blue) in the early (A) and delayed (B) aftermath, according to parents’ own PTSD classification. Panels A and B are drawn to scale in respect to annual rates, as indicated by the axis between the two panels. The width of the coloured boxes is proportional to the number of individuals within the subgroup. The corresponding pre-disaster values (white boxes) are included for reference purposes only.

Figure 2 presents observed post-disaster frequencyof GP visits in parents according to their own PTSD classification. Both mothers and fathers had an observed higher post- than pre-disaster mean frequency of GP visits, irrespective of their PTSD classification, both in the early and delayed aftermath of the terrorist attack. This was, for most cases, confirmed by bootstrap BC_a_ confidence intervals for ratios of means (Supplemental data Table 2). Notably, mean observed post-disaster frequency of GP visits increased according to parents' PTSD classification, hitting a maximum in individuals classified with full PTSD.

**Figure 3.**
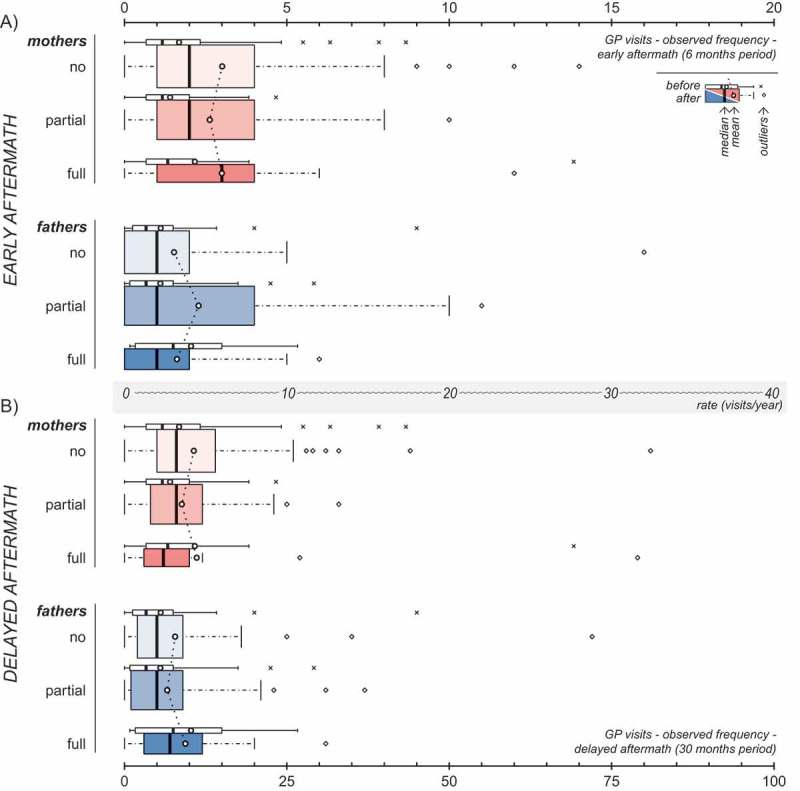
Observed post-disaster frequency of GP visits in mothers (red) and fathers (blue) in the early (A) and delayed (B) aftermath, according to the PTSD classification of their children. Panels A and B are drawn to scale in respect to annual rates, as indicated by the axis between the two panels. The width of the coloured boxes is proportional to the number of individuals within the subgroup. The corresponding pre-disaster values (white boxes) are included for reference purposes only.

Figure 3 presents observed post-disaster frequency of GP visits in parents according to PTSD classification of their surviving child. In most cases, higher post- than pre-disaster frequency of GP visits was found. However, no apparent trend related to PTSD classification was observed.

**Figure 4.**
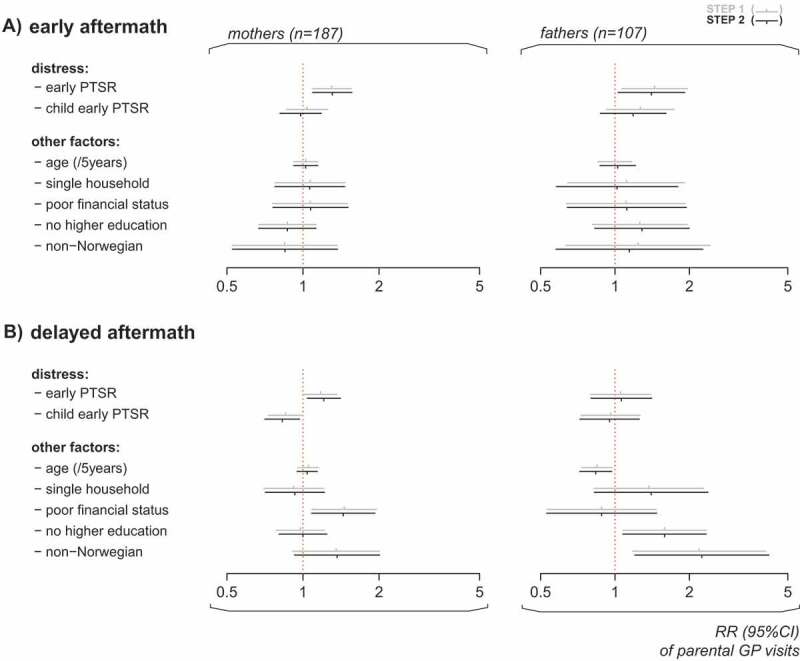
Frequency of GP visits in mothers and fathers in the early (A) and delayed (B) aftermath of the Utøya attack related to the parents’ own and their children’s early PTSR (estimated rate ratios (RR) with 95% confidence intervals). Hierarchical negative binomial regressions. Step 1: Regressions of parent and child PTSR in separate models, each adjusted for pre-disaster frequency of GP visits and socio-demography. Socio-demography shown in the chart stems from regressions of parent PTSR. Step 2: Regression of parent and child PTSR in a mutually adjusted model, including all variables from the previous step. All regressions were offset for observation time (non-admittance to hospital). Only individuals with no missing values were included. Horizontal dotted line: no relationship (RR = 1). Complete numerical figures available in Supplemental data Table 3.

Regressions of parent post-disaster frequency of GP visits related to parent and child early PTSR are presented in Figure 4. Notably, the separate models of PTSR in parent and child (Step 1) and the mutually adjusted models of PTSR in both parent and child (Step 2) returned similar conclusions and will be referred to collectively in the following. In the early aftermath of the attack, higher levels of early PTSR in parents predicted significantly higher frequency of GP visits in both mothers and fathers. In contrast, higher level of PTSR in their children did not. In the delayed aftermath, different patterns of primary healthcare consumption in mothers and fathers emerged. In mothers, higher levels of their own early PTSR predicted a significantly increased frequency of GP visits, while higher levels of their children’s early PTSR predicted the reverse. In fathers, no significant association between frequency of GP visits in the delayed aftermath and early levels of PTSR was found. Instead, lower age, lower education and non-Norwegian origin were significantly associated with increased frequency of GP visits in fathers in the delayed aftermath. Taken together, mothers’ levels of early distress significantly predicted their levels of GP visits both in the short and long term. Fathers’ early levels of distress significantly predicted their levels of GP visits shortly after the attack, while being a younger father, being less educated and of non-Norwegian origin predicted the use of GP visits in the long term. Child distress significantly predicted lower levels of GP visits only in mothers in the delayed aftermath.

**Figure 5.**
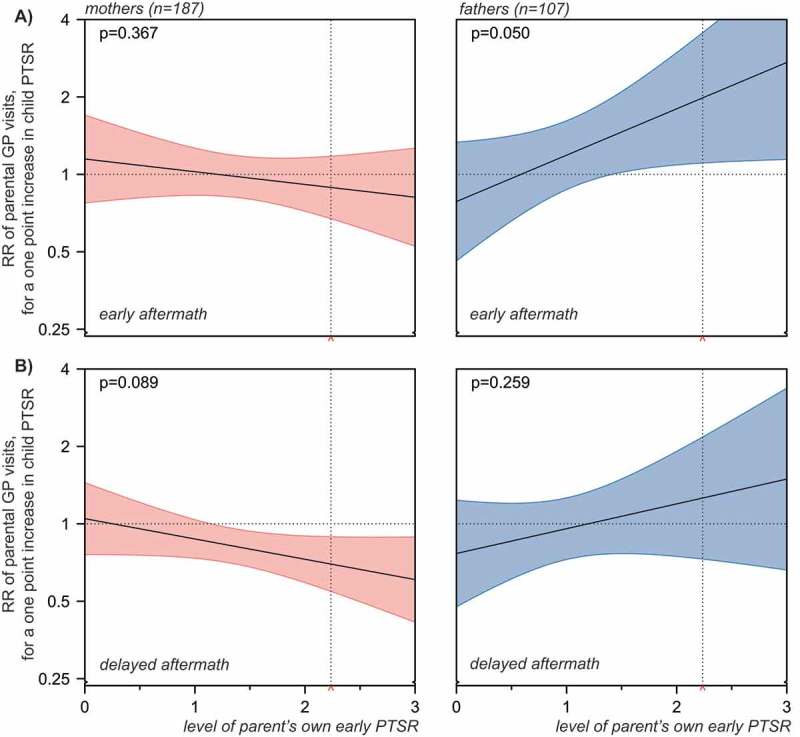
GP visits in mothers (red) and fathers (blue) related to interaction between parent and child early PTSR. The panels present the associations between frequency of parent’s GP visits and their child’s PTSR, across low through high levels of parent’s own early PTSR, in the early (A) and delayed (B) aftermath of the Utøya terrorist attack. The horizontal dotted line indicates no relationship (rate ratio = 1). The 95% confidence intervals of rate ratios for parents’ GP visits are visualized by colour shaded areas. For values of parent PTSR, where no overlap between the line of no relationship and the confidence intervals is observed, significant associations between child PTSR and the frequency of parental GP visits are indicated by the model. The vertical dotted line indicates the cut-off for probable PTSD diagnosis on the scale (mean PTSR score = 2.24, included for reference purposes only). P-values are overall estimates for interaction of each model.

Children’s distress was explored further in analyses of the interaction between child and parent PTSR as detailed in Figure 5. In neither model analysed, significant overall interactions were demonstrated (*p* = .050 for GP visits by fathers in the early aftermath, *p* = .089 for GP visits by mothers in the delayed aftermath and *p* ≥ .259 in the remaining two models). However, the following trends were observed. In the early aftermath of the attack, early PTSR in children was significantly associated with higher frequency of GP visits in fathers with high, but not with low, levels of PTSR. In the delayed aftermath of the attack, early PTSR in children was significantly associated with lower frequency of GP visits in mothers with high, but not low, levels of PTSR.

A complete overview of output from all analyses (Step 0–4) is available in Supplemental data Table 3. Notably, in all regression models, pre-disaster frequency of GP visits was significantly associated with post-disaster frequency of GP visits (*p* < .001).

## 4. Discussion

This study of the parents of the 2011 Utøya terrorist attack survivors addressed associations between frequency of parents’ post-disaster GP visits and the early PTSR endured by the parents and their surviving children. Our study demonstrated that traumatized mothers and fathers visited their GPs significantly more frequently than did their less affected peers in the first six months following the attack. Thus, our findings indicate an important potential role of GPs in identifying healthcare needs in distressed mothers and fathers early post disaster. The GPs need to be aware that traumatized parents approach them soon after a disaster, and that this is an important window of opportunity for reaching out to them. Parenting can be challenging in the wake of disaster. A wide range of health complaints and health related challenges, such as accessing social benefits and welfare services, may trouble mothers and fathers as they struggle to regain normality. Post-disaster, distress may present both as somatic and mental health complaints (Dorn et al., 2007). Inexperienced providers may easily overlook the complexity of healthcare needs in individuals indirectly exposed to disaster, such as parents. In order for GPs to fulfil their important potential role in managing parents’ healthcare needs in the early aftermath of disaster, the primary healthcare services need to prepare adequately for identifying post-disaster healthcare needs, providing appropriate evidence-based services and for making timely referrals.

Early PTSR potentially develop into chronic disease. PTSD may trouble individuals for years (Steinert, Hofmann, Leichsenring, & Kruse, 2015). Furthermore, traumatization may elicit a range of long-term somatic health complaints, including respiratory, gastrointestinal and cardiovascular disease (Boscarino, 2004; Pacella, Hruska, & Delahanty, 2013; Schnurr, Green, & Kaltman, 2007). Thus, GPs may continue to play an important role in monitoring and providing for traumatized parents, well beyond the early aftermath of a disaster. In line with this notion, own early distress was found to be significantly associated with lasting higher frequency of GP visits in mothers. In contrast, in fathers, no significant association was demonstrated in the delayed aftermath. Insufficient power of the latter analysis or the fact that distressed fathers may have had healthcare needs covered by other providers, such as specialized healthcare providers, may speak to this finding. An alternative interpretation is that distressed fathers, more than mothers, were reluctant to seek help for potential mental health issues, as has been found in several previous studies (Addis & Mahalik, 2003; Oliver, Pearson, Coe, & Gunnell, 2005). Post-disaster primary healthcare of fathers remains to be further investigated.

Healthcare needs in parents may reflect more than ill-health of the individual mother or father. Parents are the principal providers of care to their children, including adolescents and young adults (Grills-Taquechel, Littleton, & Axsom, 2011). Distress of traumatized children may directly influence healthcare needs in parents. Surprisingly, in our study, no significant overall increase in parents’ frequency of GP visits related to survivors’ early PTSR was found, neither in models adjusted nor unadjusted for parents’ own early PTSR. On the contrary in mothers, higher levels of early PTSR in the survivors predicted significantly fewer GP visits among mothers in the delayed aftermath of the terrorist attack. One possible explanation is that mothers accompanying their children to healthcare services may have benefitted from such visits or been included in family treatment approaches. Thus, needs for primary healthcare services may potentially have been lower in mothers of the most distressed survivors. An alternative explanation is that mothers of troubled children may have focused on helping their child and potentially neglected their own healthcare needs.

We found no significant overall interactions. However, we observed some associations between child PTSR and frequency of parental GP visits. The associations differed between mothers and fathers and between the early and delayed aftermath, and should be interpreted with caution. Indications that the child’s stress reactions may impact help seeking mainly in distressed parents, perhaps differently for mothers and fathers, need further exploration in future research before conclusions can be drawn.

Review of previous literature has suggested significantly higher post-trauma general healthcare consumption in adults experiencing higher levels of posttraumatic distress than in their peers experiencing lower levels of posttraumatic distress (Elhai et al., 2005), in line with our findings. However, previous literature may not directly compare to our study. Firstly, previous research on post-disaster healthcare consumption and PTSR in traumatized adults has been limited to adult survivors of trauma. To our knowledge, no studies have hitherto addressed post-disaster healthcare consumption related to self-reported distress symptoms in parents experiencing life threat to their child. Secondly, previous studies of adult survivors have largely analysed self-reported healthcare consumption, oftenwith dichotomous healthcare measures, rather than the observed frequencies of visits. Thirdly, few studies include information on pre-disaster healthcare consumption. In our study, all analyses were adjusted for pre-disaster frequency of GP visits, age, level of education, financial status, country of origin, whether living alone or with a partner and PTSR of the child survivor. Thus, our findings demonstrate not only a significant association between parent distress and frequency of early post-disaster GP visits, but also that the associations appeared independently of adjustment for pre-disaster primary healthcare consumption, sociodemographic factors and the PTSR of their child survivor.

### 4.1. Strengths and limitations

This study combines registry-based healthcare data with self-reported distress in the mothers, fathers and their surviving children. We had the advantage of an objective outcome measure of healthcare consumption, as well as a detailed, subjective account of PTSR endured by the participants. Thus, uniquely, the study allowed for assessment of parents’ healthcare consumption related to PTSR in a family context and the adjustment of analyses for participants’ pre-disaster health-seeking behaviours. Pre- and post-disaster frequency of GP visits were assessed using administrative claims data that had been routinely reported by the GPs at the time of service provision. Thus, recall bias on behalf of the patient or the provider was eliminated. However, we cannot rule out aberrations in the GP records. For example, joint consultations of parents and children may erroneously have been registered as child or parent consultations only. Self-reports of PTSR were obtained from participants in Wave 1 of the study. As PTSR may have a fluctuating trajectory, cross-sectional assessment at the time of Wave 1 may not accurately represent the level of distress experienced throughout the early time period. Moreover, parent and child PTSR were measured on the same scale. However, as the scale was originally developed for investigations of adolescent samples, it has not been validated in adult populations.

In this study, the Utøya terrorist attack serves as a model for parent traumatization through indirect exposure to life threat to a child. The terrorist attack occurred within a limited time and space and the survivors were fairly homogenously exposed to the terrorist threat. Furthermore, none of the parents were in the proximity of the island at the time of attack nor were they threatened by the perpetrator. Thus, all parents were exposed to the terrorist threat exclusively through learning of the event. However, it should be noted that the 2011 Utøya terrorist attack happened in the aftermath of another terrorist attack, a bomb in the governmental quarter in Oslo, earlier the same day. Devastation, chaos and uncertainty following this blast may have added to the distress of some parents, in particular parents present in Oslo at the time.

Following the Utøya terrorist attack, primary healthcare was available across affected municipalities throughout the country, with few restrictions to the capacity of GPs to deliver care. We do not know to what extent the early proactive outreach programme influenced post-disaster doctor-seeking behaviours. The early proactive outreach programme may both have increased frequency of GP visits, by channelling more people to the services, as well as decreased the frequency of GP visits, by solving simple problems without the involvement of the regular healthcare services. In the minority of parents having their GP as a dedicated contact person, outreach services may have been reported as regular GP consultations to the HELFO database and hence included in our analyses. In Norway, referrals are obligatory for accessing specialized healthcare services. However, we may not rule out that some parents, due to the unprecedented nature of the terrorist attack, may have bypassed their GP and the regular referral system, e.g. with the help of their contact person in the municipality. Furthermore, irrespectively of mode of referral, individuals accessing specialized healthcare services may have had less of a need for primary healthcare services, as their primary healthcare needs may in part have been covered by their specialist provider. Finally, frequent GP visits are no guarantee for PTSR being recognized, let alone adequately provided for.

The ordered aftermath of the Utøya terrorist attack allowed for complete identification of all survivors at the time they were rescued from the island. The response rate among the survivors was high. Identifying the parent population was more challenging due to the diversity of modern family arrangements. However, unlike most studies of child and youth survivors, we were able to recruit both the mothers and fathers of a majority of the survivor participants. Nevertheless, despite the survivor population being known, the full size of the parent population remains unknown; survivors may have had additional caregivers to those identified by this study. Our analyses did not address cohabitation of parent and child. In survivors of this age group, a flexible and changeable cohabitation with parents may be expected. Youth may need to move temporarily or permanently to schools, universities or new workplaces. Nearly half of the parents of this cohort were divorced and sustained separate households (Haga et al., 2015). Thus, despite knowing that 60–70% of the survivors lived with one or more of their parents (Stene & Dyb, 2016), we had insufficiently accurate information on which of their parents they lived with and if they split their time between their different homes.

Our findings may be most applicable to comparable trauma and healthcare contexts. Whether our findings may be generalizable to healthcare needs that may arise in parents following life threat to offspring in the context of terrorism or natural disasters, accidents or serious illness of a child  remains to be investigated.

All analyses were correlational. No causality can be demonstrated. We made no assessment of content or efficacy of the healthcare services provided.

### 4.2. Conclusion

Our study suggests that GPs may play a critical role both in identifying and in providing for the needs of traumatized parents of disaster survivors. GPs need to be aware that distressed individuals may be more likely to come to them in the aftermath of a disaster, and that this may be an important window in which to offer care. GPs must prepare for both short- and long-term healthcare needs. Distressed mothers of traumatized survivors may be at increased risk of being underserved in the delayed aftermath of a disaster, calling for the particular attention from GPs.

## Supplementary Material

Supplementary material

## Availability of data and material

Data supporting the findings of this study are not publicly available. Self-reports may be accessed through the Norwegian Centre for Violence and Traumatic Stress Studies (NKVTS). Administrative claims data on primary healthcare consumption and data from the Norwegian Patient Registry (NPR) may be accessed through the Norwegian Directorate of Health (https://helsedirektoratet.no/english). Restrictions apply. Technical appendix and statistical code are available from the corresponding author.

## References

[CIT0001] Addis M. E., & Mahalik J. R. (2003). Men, masculinity, and the contexts of help seeking. *The American Psychologist*, 58(1), 5–13. 12674814 10.1037/0003-066x.58.1.5

[CIT0002] American Psychiatric Association (1994). *Diagnostic and statistical manual of mental disorders* (4th ed.). Washington, DC: APA.

[CIT0003] Andersen R. M. (1995). Revisiting the behavioral model and access to medical care: Does it matter?. *Journal of Health and Social Behavior*, 36(1), 1–10. 7738325

[CIT0004] Boscarino J. A. (2004). Posttraumatic stress disorder and physical illness: Results from clinical and epidemiologic studies. *Annals of the New York Academy of Sciences*, 1032, 141–153. doi:10.1196/annals.1314.011 15677401

[CIT0005] Brewin C. R., Fuchkan N., Huntley Z., Robertson M., Thompson M., Scragg P., … Ehlers A. (2010). Outreach and screening following the 2005 London bombings: Usage and outcomes. *Psychological Medicine*, 40(12), 2049–2057. doi:10.1017/s0033291710000206 20178677 PMC2964043

[CIT0006] Bugge I., Dyb G., Stensland S. Ø., Ekeberg Ø., Wentzel-Larsen T., & Diseth T. H. (2015). Physical injury and posttraumatic stress reactions. A study of the survivors of the 2011 shooting massacre on Utøya Island, Norway. *Journal of Psychosomatic Research*, 79(5), 384–390. doi:10.1016/j.jpsychores.2015.09.005 26526313

[CIT0007] Cabizuca M., Marques-Portella C., Mendlowicz M. V., Coutinho E. S., & Figueira I. (2009). Posttraumatic stress disorder in parents of children with chronic illnesses: A meta-analysis. *Health Psychology*, 28(3), 379–388. doi:10.1037/a0014512 19450045

[CIT0008] Dorn T., Yzermans J. C., Spreeuwenberg P. M., & Van der Zee J. (2007). Physical and mental health problems in parents of adolescents with burns–a controlled, longitudinal study. *Journal of Psychosomatic Research*, 63(4), 381–389. doi:10.1016/j.jpsychores.2007.02.005 17905046

[CIT0009] Dyb G., Holen A., Steinberg A. M., Rodriguez N., & Pynoos R. S. (2003). Alleged sexual abuse at a day care center: Impact on parents. *Child Abuse & Neglect*, 27(8), 939–950. 12951142 10.1016/s0145-2134(03)00141-8

[CIT0010] Dyb G., Jensen T., Glad K. A., Nygaard E., & Thoresen S. (2014). Early outreach to survivors of the shootings in Norway on the 22nd of July 2011. *European Journal of Psychotraumatology*, 5, 23523. doi:10.3402/ejpt.v5.23523 PMC4082194 25018858

[CIT0011] Elhai J. D., Calhoun P. S., & Ford J. D. (2008). Statistical procedures for analyzing mental health services data. *Psychiatry Research*, 160(2), 129–136. doi:10.1016/j.psychres.2007.07.003 18585790

[CIT0012] Elhai J. D., North T. C., & Frueh B. C. (2005). Health service use predictors among trauma survivors: A critical review. *Psychological Services*, 2(1), 3–19. doi:10.1037/1541-1559.2.1.3

[CIT0013] Gavrilovic J. J., Schützwohl M., Fazel M., & Priebe S. (2005). Who seeks treatment after a traumatic event and who does not? A review of findings on mental health service utilization. *Journal of Traumatic Stress*, 18(6), 595–605. doi:10.1002/jts.20068 16382432

[CIT0014] Grills-Taquechel A. E., Littleton H. L., & Axsom D. (2011). Social support, world assumptions, and exposure as predictors of anxiety and quality of life following a mass trauma. *Journal of Anxiety Disorders*, 25(4), 498–506. doi:10.1016/j.janxdis.2010.12.003 21236630 PMC3074027

[CIT0015] Haga J. M., Stene L. E., Wentzel-Larsen T., Thoresen S., & Dyb G. (2015). Early postdisaster health outreach to modern families: A cross-sectional study. *BMJ Open*, 5(12), e009402. doi:10.1136/bmjopen-2015-009402 PMC4691779 26681694

[CIT0016] Kantor V., Knefel M., & Lueger-Schuster B. (2016). Perceived barriers and facilitators of mental health service utilization in adult trauma survivors: A systematic review. *Clinical Psychology Review*, 52, 52–68. doi:10.1016/j.cpr.2016.12.001 28013081

[CIT0017] Nelson L. P., & Gold J. I. (2012). Posttraumatic stress disorder in children and their parents following admission to the pediatric intensive care unit: A review. *Pediatric Critical Care Medicine*, 13(3), 338–347. doi:10.1097/PCC.0b013e3182196a8f 21499173

[CIT0018] Oliver M. I., Pearson N., Coe N., & Gunnell D. (2005). Help-seeking behaviour in men and women with common mental health problems: Cross-sectional study. *The British Journal of Psychiatry*, 186, 297–301. doi:10.1192/bjp.186.4.297 15802685

[CIT0019] Pacella M. L., Hruska B., & Delahanty D. L. (2013). The physical health consequences of PTSD and PTSD symptoms: A meta-analytic review. *Journal of Anxiety Disorders*, 27(1), 33–46. doi:10.1016/j.janxdis.2012.08.004 23247200

[CIT0020] Ringard Å., Sagan A., Sperre Saunes I., & Lindahl A. K. (2013). Norway: Health system review. *Health Systems in Transition*, 15(8), 1–162. 24434287

[CIT0021] Rodriguez J. J., & Kohn R. (2008). Use of mental health services among disaster survivors. *Current Opinion in Psychiatry*, 21(4), 370–378. doi:10.1097/YCO.0b013e328304d984 18520742

[CIT0022] Røkholt E. G., Schultz J.-H., & Langballe Å. (2016). Negotiating a new day: Parents’ contributions to supporting students’ school functioning after exposure to trauma. *Psychology Research and Behavior Management*, 9, 81–93. doi:10.2147/prbm.s97229 27175097 PMC4854255

[CIT0023] Schnurr P. P., Green B. L., & Kaltman S. (2007). *Trauma exposure and physical health*. New York, NY: Guilford Press.

[CIT0024] Scrimin S., Axia G., Capello F., Moscardino U., Steinberg A. M., & Pynoos R. S. (2006). Posttraumatic reactions among injured children and their caregivers 3 months after the terrorist attack in Beslan. *Psychiatry Research*, 141(3), 333–336. doi:10.1016/j.psychres.2005.11.004 16515809

[CIT0025] Steinberg A. M., Brymer M. J., Decker K. B., & Pynoos R. S. (2004). The University of California at Los Angeles post-traumatic stress disorder reaction index. *Current Psychiatry Reports*, 6(2), 96–100. 15038911 10.1007/s11920-004-0048-2

[CIT0026] Steinert C., Hofmann M., Leichsenring F., & Kruse J. (2015). The course of PTSD in naturalistic long-term studies: High variability of outcomes. A systematic review. *Nordic Journal of Psychiatry*, 69(7), 483–496. doi:10.3109/08039488.2015.1005023 25733025

[CIT0027] Stene L. E, & Dyb G. (2016). Research participation after terrorism: an open cohort study of survivors and parents after the 2011 utøya attack in norway. *Bmc Res Notes*, 9, 57. doi: 10.1186/s13104-016-1873-1 26830191 PMC4736239

[CIT0028] The Norwegian Directorate of Health (2012). *Statistics of regular general practitoners 2011*. Oslo: Author Retrieved from http://helsedirektoratet.no/finansiering/refusjonsordninger/tall-og-analyser/fastlege/Sider/fastlegestatistikken-2011.aspx

[CIT0029] Thoresen S., Jensen T. K., Wentzel-Larsen T., & Dyb G. (2016). Parents of terror victims. A longitudinal study of parental mental health following the 2011 terrorist attack on Utøya Island. *Journal of Anxiety Disorders*, 38, 47–54. doi:10.1016/j.janxdis.2016.01.004 26812593

