# Supplementary material for: Does posttraumatic stress predict frequency of general practitioner visits in parents of terrorism survivors? A longitudinal study
Source: Eur J Psychotraumatol. 2017 Nov 20;8(1):1389206. doi: 10.1080/20008198.2017.1389206 (PMC5784312; doi:10.1080/20008198.2017.1389206)
Supplement: Supplementary material [file ZEPT_A_1389206_SM7243.docx]

**Supplementary Table 1:** Regressions of parent attrition, related to (a) sociodemograpy (analysed in the full parent cohort, n=532 parents), and to (b) early posttraumatic stress reactions (PTSR) (analysed in parent participants, whose participation included Wave 1, n=453 parents). Logistic regressions, odds ratio (OR) with 95% confidence intervals. Clustering of members of the same families was addressed by the gee-procedure with an exchangeable correlation structure in the regression analyses. Sociodemography for these analyses was derived from all three waves of data collection. Discrepancy between the waves was resolved by using the first value reported.

| **a)** | Unadjusted |  |  | Adjusted^1^ |  |
| --- | --- | --- | --- | --- | --- |
| n=523 parents^2^ | OR (95%CI) | p |  | OR (95%CI) | p |
| male gender | 1.96 (1.47-2.60) | < 0.001 |  | 1.96 (1.44-2.69) | < 0.001 |
| age (/5years) | 1.08 (0.93-1.25) | 0.302 |  | 0.98 (0.83-1.15) | 0.766 |
| single parent | 0.83 (0.51-1.34) | 0.441 |  | 1.06 (0.62-1.80) | 0.841 |
| no higher education | 0.99 (0.70-1.41) | 0.984 |  | 1.00 (0.70-1.41) | 0.981 |
| poor financial status | 0.54 (0.28-1.02) | 0.057 |  | 0.56 (0.29-1.10) | 0.091 |
| non-Norwegian origin | 0.69 (0.34-1.29) | 0.246 |  | 0.72 (0.37-1.39) | 0.766 |

^1^ Adjusted regressions were adjusted for all variables shown.

^2^ One or more missing variables were found in nine parents, who were consequently not included in the analyses (1.7%).

| **b)** | Unadjusted |  |  | Adjusted^1^ |  |
| --- | --- | --- | --- | --- | --- |
| n=447 parents^2^ | OR (95%CI) | p |  | OR (95%CI) | p |
| PTSR | 0.70 (0.51-0.95) | 0.023 |  | 0.88 (0.64-1.20) | 0.409 |

^1^ Adjusted regressions were adjusted for gender, age, whether living alone or with a partner, higher education, financial status and country of origin (variables not shown).

^2^ One or more missing variables were found in six parents, who were consequently not included in the analyses (1.3%).

**Supplementary Table 2:** Pairwise comparison of frequency of parents’ GP visits before and after the terrorist attack according to parent’s and child’s posttraumatic stress disorder (PTSD) classification. RR= rate ratio, mean after by before disaster estimates. CI= bias-corrected and accelerated (BC_a_) confidence intervals from bootstraps of 10,000 replications.

|  | mothers (n=196) | |  | fathers (n=112) | |
| --- | --- | --- | --- | --- | --- |
|  | rate,  before/after | RR (95%CI) |  | rate,  before/after | RR (95%CI) |
| EARLY AFTERMATH |  |  |  |  |  |
| *Parent distress* |  |  |  |  |  |
| - no PTSD | 2.82/4.88 | 1.73 (1.45-2.06) |  | 2.40/3.30 | 1.38 (1.10-1.68) |
| - partial PTSD | 3.61/6.91 | 1.91 (1.48-2.33) |  | 2.31/4.67 | 2.02 (0.87-3.58) |
| - full PTSD | 4.35/8.00 | 1.84 (1.20-2.85) |  | 4.11/10.67 | 2.59^a^ |
|  |  |  |  |  |  |
| *Child distress* |  |  |  |  |  |
| - no PTSD | 3.31/5.94 | 1.79 (1.48-2.17) |  | 2.31/3.22 | 1.39 (1.04-1.79) |
| - partial PTSD | 2.85/5.38 | 1.89 (1.55-2.29) |  | 2.10/4.27 | 2.04 (1.48-2.78) |
| - full PTSD | 4.54/6.00 | 1.32 (0.92-2.13) |  | 4.42/3.33 | 0.75 (0.34-1.50) |
|  |  |  |  |  |  |
| DELAYED AFTERMATH |  |  |  |  |  |
| *Parent distress* |  |  |  |  |  |
| - no PTSD | 2.82/3.34 | 1.18 (1.00-1.55) |  | 2.40/2.82 | 1.18 (0.96-1.46) |
| - partial PTSD | 3.61/4.67 | 1.29 (1.10-1.54) |  | 2.31/3.37 | 1.46 (1.06-2.15) |
| - full PTSD | 4.35/6.16 | 1.41 (0.98-2.05) |  | 4.11/7.87 | 1.91^a^ |
|  |  |  |  |  |  |
| *Child distress* |  |  |  |  |  |
| - no PTSD | 3.31/4.23 | 1.28 (1.06-1.67) |  | 2.31/3.18 | 1.38 (1.10-1.81) |
| - partial PTSD | 2.85/3.53 | 1.24 (1.06-1.46) |  | 2.10/2.44 | 1.16 (0.84-1.59) |
| - full PTSD | 4.54/4.65 | 1.02 (0.68-1.82) |  | 4.42/4.07 | 0.92 (0.53-1.61) |
| ^a^ insufficient number of cases for reliable bootstrap | | | | | |

**Supplementary Table 3:** Frequency of GP visits in mothers and fathers in the early (A) and delayed (B) aftermath of the Utøya attack related to parents’ own and their children’s PTSR (estimated rate ratios, RR, with 95% confidence intervals). This table supplements Figure 4. Hierarchical negative binomial regressions: Step 0: Regressions of parent and child PTSR in separate models (A and B), each adjusted for pre-disaster GP visits. Step 1: Regressions of parent and child PTSR in separate models (A and B), each adjusted for parent’s pre-disaster GP visits and sociodemography. Step 2: Regression of parent and child PTSR in one common, mutually adjusted model that included all variables of the previous step. Step 3: Regression of parent and child PTSR, and of the interaction between the two, in one common, mutually adjusted model that included all variables of the previous step. All analyses were offset for observation time (non-admittance to hospital).

|  |  | |  | |
| --- | --- | --- | --- | --- |
|  |  | |  | |
|  | **Mothers (n=187)** | | **Fathers (n=107)** | |
| **STEP 0A** | RR (95% CI) | p | RR (95% CI) | p |
| *early aftermath* |  |  |  |  |
| - parent’s early PTSR | 1.27 (1.08-1.50) | 0.004 | 1.49 (1.09-2.02) | 0.011 |
| - pre-disaster GP visits | 1.04 (1.03-1.06) | <0.001 | 1.06 (1.04-1.08) | <0.001 |
|  |  |  |  |  |
| *delayed aftermath* |  |  |  |  |
| - parent’s early PTSR | 1.22 (1.06-1.42) | 0.007 | 1.11 (0.83-1.49) | 0.471 |
| - pre-disaster GP visits | 1.04 (1.03-1.05) | <0.001 | 1.06 (1.04-1.09) | <0.001 |
|  |  |  |  |  |
|  |  |  |  |  |
| **STEP 0B** | RR (95% CI) | p | RR (95% CI) | p |
| *early aftermath* |  |  |  |  |
| - child’s early PTSR | 1.04 (0.87-1.26) | 0.661 | 1.24 (0.93-1.67) | 0.148 |
| - pre-disaster GP visits | 1.05 (1.04-1.06) | <0.001 | 1.06 (1.04-1.09) | <0.001 |
|  |  |  |  |  |
| *delayed aftermath* |  |  |  |  |
| - child’s early PTSR | 0.86 (0.73-1.01) | 0.067 | 1.12 (0.86-1.47) | 0.397 |
| - pre-disaster GP visits | 1.04 (1.03-1.05) | <0.001 | 1.06 (1.04-1.08) | <0.001 |
|  |  |  |  |  |
|  |  |  |  |  |
| **STEP 1A** | RR (95% CI) | p | RR (95% CI) | p |
| *early aftermath* | |  |  |  |
| - parent’s early PTSR | 1.30 (1.09-1.55) | 0.003 | 1.44 (1.06-1.96) | 0.019 |
| - age (/5years) | 1.03 (0.92-1.14) | 0.656 | 1.00 (0.85-1.17) | 0.976 |
| - single parent | 1.07 (0.78-1.47) | 0.689 | 1.11 (0.65-1.91) | 0.703 |
| - poor financial status | 1.07 (0.76-1.50) | 0.709 | 1.11 (0.64-1.92) | 0.718 |
| - no higher education | 0.87 (0.67-1.13) | 0.289 | 1.26 (0.81-1.96) | 0.304 |
| - non-Norwegian | 0.85 (0.52-1.36) | 0.492 | 1.24 (0.63-2.43) | 0.532 |
| - number of surviving children | 1.19 (0.72-1.97) | 0.502 | 0.68 (0.20-2.29) | 0.539 |
| - pre-disaster GP visits | 1.04 (1.03-1.06) | <0.001 | 1.05 (1.03-1.08) | <0.001 |
|  |  |  |  |  |
| *delayed aftermath* | |  |  |  |
| - parent’s early PTSR | 1.17 (1.01-1.36) | 0.036 | 1.05 (0.79-1.39) | 0.723 |
| - age (/5years) | 1.05 (0.96-1.16) | 0.283 | 0.85 (0.73-0.98) | 0.021 |
| - single parent | 0.92 (0.70-1.21) | 0.544 | 1.37 (0.82-2.29) | 0.225 |
| - poor financial status | 1.46 (1.09-1.95) | 0.012 | 0.88 (0.53-1.47) | 0.631 |
| - no higher education | 0.98 (0.78-1.22) | 0.829 | 1.59 (1.08-2.35) | 0.019 |
| - non-Norwegian | 1.35 (0.91-2.00) | 0.133 | 2.19 (1.18-4.06) | 0.013 |
| - number of surviving children | 0.88 (0.56-1.38) | 0.582 | 0.57 (0.20-1.66) | 0.304 |
| - pre-disaster GP visits | 1.03 (1.02-1.05) | <0.001 | 1.06 (1.04-1.08) | <0.001 |
|  |  |  |  |  |
|  |  |  |  |  |
| **STEP 1B** | RR (95% CI) | p | RR (95% CI) | p |
| *early aftermath* | |  |  |  |
| - child’s early PTSR | 1.04 (0.86-1.25) | 0.706 | 1.27 (0.92-1.74) | 0.145 |
| - age (/5years) | 1.00 (0.90-1.13) | 0.908 | 1.05 (0.88-1.24) | 0.606 |
| - single parent | 1.06 (0.77-1.47) | 0.717 | 1.13 (0.64-2.01) | 0.262 |
| - poor financial status | 1.11 (0.78-1.57) | 0.568 | 1.08 (0.61-1.91) | 0.795 |
| - no higher education | 0.85 (0.77-1.12) | 0.717 | 1.30 (0.82-2.04) | 0.262 |
| - non-Norwegian | 0.99 (0.62-1.59) | 0.966 | 1.28 (0.64-2.58) | 0.490 |
| - number of surviving children | 1.11 (0.67-1.86) | 0.678 | 0.57 (0.16-1.99) | 0.379 |
| - pre-disaster GP visits | 1.05 (1.03-1.06) | <0.001 | 1.05 (1.03-1.08) | <0.001 |
|  |  |  |  |  |
| *delayed aftermath* | |  |  |  |
| - child’s early PTSR | 0.85 (0.73-1.00) | 0.049 | 0.96 (0.73-1.26) | 0.773 |
| - age (/5years) | 1.02 (0.93-1.12) | 0.676 | 0.84 (0.72-0.97) | 0.020 |
| - single parent | 0.93 (0.71-1.23) | 0.624 | 1.43 (0.84-2.42) | 0.186 |
| - poor financial status | 1.50 (1.12-2.01) | 0.006 | 0.89 (0.53-1.47) | 0.639 |
| - no higher education | 0.98 (0.79-1.22) | 0.868 | 1.58 (1.07-2.33) | 0.022 |
| - non-Norwegian | 1.56 (1.06-2.29) | 0.024 | 2.30 (1.24-4.29) | 0.008 |
| - number of surviving children | 0.84 (0.54-1.31) | 0.434 | 0.57 (0.20-1.65) | 0.299 |
| - pre-disaster GP visits | 1.04 (1.02-1.05) | <0.001 | 1.06 (1.04-1.08) | <0.001 |
|  |  |  |  |  |
|  |  |  |  |  |
|  |  |  |  |  |
|  |  |  |  |  |
| **STEP 2** | RR (95% CI) | p | RR (95% CI) | p |
| *early aftermath* | |  |  |  |
| - parent’s early PTSR | 1.31 (1.09-1.56) | 0.003 | 1.40 (1.03-1.91) | 0.032 |
| - child’s early PTSR | 0.98 (0.81-1.18) | 0.822 | 1.18 (0.87-1.61) | 0.277 |
| - age (/5years) | 1.02 (0.92-1.14) | 0.662 | 1.03 (0.87-1.21) | 0.760 |
| - single parent | 1.06 (0.77-1.46) | 0.701 | 1.02 (0.58-1.80) | 0.946 |
| - poor financial status | 1.07 (0.76-1.51) | 0.693 | 1.12 (0.64-1.94) | 0.697 |
| - no higher education | 0.87 (0.67-1.13) | 0.286 | 1.29 (0.83-2.00) | 0.263 |
| - non-Norwegian | 0.85 (0.53-1.37) | 0.501 | 1.14 (0.58-2.27) | 0.702 |
| - number of surviving children | 1.19 (0.72-1.97) | 0.496 | 0.65 (0.19-2.18) | 0.486 |
| - pre-disaster GP visits | 1.04 (1.03-1.06) | <0.001 | 1.05 (1.03-1.08) | <0.001 |
|  |  |  |  |  |
| *delayed aftermath* | |  |  |  |
| - parent’s early PTSR | 1.21 (1.04-1.41) | 0.013 | 1.06 (0.80-1.41) | 0.682 |
| - child’s early PTSR | 0.83 (0.71-0.97) | 0.019 | 0.95 (0.72-1.25) | 0.720 |
| - age (/5years) | 1.04 (0.95-1.14) | 0.413 | 0.84 (0.72-0.97) | 0.019 |
| - single parent | 0.93 (0.71-1.22) | 0.590 | 1.40 (0.83-2.38) | 0.212 |
| - poor financial status | 1.44 (1.08-1.93) | 0.013 | 0.88 (0.53-1.47) | 0.634 |
| - no higher education | 1.00 (0.80-1.24) | 0.996 | 1.59 (1.08-2.34) | 0.020 |
| - non-Norwegian | 1.36 (0.93-2.01) | 0.115 | 2.25 (1.20-4.19) | 0.011 |
| - number of surviving children | 0.90 (0.58-1.39) | 0.625 | 0.58 (0.20-1.69) | 0.320 |
| - pre-disaster GP visits | 1.03 (1.02-1.04) | <0.001 | 1.06 (1.04-1.08) | <0.001 |
|  |  |  |  |  |
| **STEP 3** | RR (95% CI) | p | RR (95% CI) | p |
| *early aftermath* | |  |  |  |
| - parent’s early PTSR | 1.56 (1.01-2.41) | 0.045 | 0.71 (0.33-1.52) | 0.377 |
| - child early PTSR | 1.15 (0.77-1.70) | 0.493 | 0.78 (0.46-1.33) | 0.369 |
| - PTSR parent/child interaction | 0.89 (0.70-1.14) | 0.367 | 1.51 (1.00-2.29) | 0.050 |
| - age (/5years) | 1.03 (0.92-1.15) | 0.648 | 1.01 (0.86-1.19) | 0.872 |
| - single parent | 1.06 (0.77-1.46) | 0.709 | 0.89 (0.51-1.56) | 0.680 |
| - poor financial status | 1.10 (0.78-1.55) | 0.599 | 0.97 (0.56-1.68) | 0.915 |
| - no higher education | 0.87 (0.67-1.13) | 0.284 | 1.27 (0.82-1.96) | 0.282 |
| - non-Norwegian | 0.86 (0.54-1.39) | 0.542 | 1.04 (0.53-2.05) | 0.912 |
| - number of surviving children | 1.23 (0.74-2.03) | 0.424 | 0.71 (0.22-2.29) | 0.563 |
| - pre-disaster GP visits | 1.04 (1.03-1.06) | <0.001 | 1.05 (1.03-1.08) | <0.001 |
|  |  |  |  |  |
| *delayed aftermath* | |  |  |  |
| - parent’s early PTSR | 1.62 (1.12-2.33) | 0.010 | 0.75 (0.38-1.47) | 0.409 |
| - child early PTSR | 1.05 (0.76-1.45) | 0.778 | 0.77 (0.48-1.24) | 0.275 |
| - PTSR parent/child interaction | 0.83 (0.68-1.03) | 0.089 | 1.25 (0.85-1.83) | 0.259 |
| - age (/5years) | 1.04 (0.95-1.14) | 0.422 | 0.84 (0.72-0.97) | 0.020 |
| - single parent | 0.92 (0.71-1.21) | 0.565 | 1.28 (0.75-2.18) | 0.364 |
| - poor financial status | 1.50 (1.12-2.00) | 0.006 | 0.83 (0.49-1.39) | 0.480 |
| - no higher education | 0.98 (0.79-1.22) | 0.872 | 1.56 (1.06-2.30) | 0.025 |
| - non-Norwegian | 1.38 (0.94-2.03) | 0.099 | 2.05 (1.10-3.82) | 0.024 |
| - number of surviving children | 0.93 (0.60-1.45) | 0.746 | 0.61 (0.21-1.74) | 0.354 |
| - pre-disaster GP visits | 1.03 (1.02-1.05) | <0.001 | 1.06 (1.04-1.08) | <0.001 |
